# Cells in Cardiovascular Disease: Using Diversity to Confront Adversity

**DOI:** 10.3390/cells9102192

**Published:** 2020-09-29

**Authors:** José Martínez-González, Pablo García de Frutos

**Affiliations:** 1 Institute for Biomedical Research of Barcelona, IIBB-CSIC, 08036 Barcelona, Spain; 2 CIBER de Enfermedades Cardiovasculares (CIBERCV), Instituto de Salud Carlos III, 28029 Madrid, Spain; 3 Institut d’Investigacions Biomèdiques Sant Pau (IIB Sant Pau), 08041 Barcelona, Spain; 4 Institut d’Investigacions Biomèdiques August Pi i Sunyer (IDIBAPS), 08036 Barcelona, Spain

**Keywords:** cardiovascular diseases, heart failure, atherosclerosis, (myo)fibroblasts, endothelium, leukocytes, vascular smooth muscle cells, myocytes

The present Special Issue on “Cells in Cardiovascular Disease” wants to offer a general overview of current cardiovascular research and illustrate how advances in the molecular characterization at the cellular level are providing unique insights into pathologies of the circulatory system. The mechanisms of response to injury and the adaptation of cells to disease processes are now being characterized in minute detail, and new paths of intervention are provided by research on the basic processes of cellular response to damage. Cardiovascular research has witnessed how the study of the cell types that make up the heart, vessels, and blood has revealed their plasticity, new cellular interactions and interconnections, as well as surprising cellular functions in certain pathophysiological settings. In the present volume, we have edited more than 25 contributions, including experimental research articles and reviews covering a broad array of research topics in cardiovascular disease (CVD). Due to the wide field covered, the articles have varied interests, ranging from molecular characterizations of pathways involved in disease to clinical observations, and from acute diseases to mechanisms of chronic remodeling of the heart and the vasculature.

Several articles focused on cardiac remodeling and chronic heart failure, a life-threatening and increasingly common condition whose study should be considered a global health priority [1]. In in vitro and in vivo experimental models, Kutsche et al. study the role of mitochondrial uncoupling protein 2 (UCP-2) in the oxidative glucose metabolism during the transition from early heart hypertrophy to late heart failure stages [2]. The silencing of UCP-2 improved the oxidative glucose metabolism. A rat model of pressure overload mirrored the effect of UCP-2 on the disease (low in compensatory hypertrophy, and increased in decompensatory state). Similarly, the authors found a correlation in samples from end-stage heart failure biopsies, suggesting that the inhibition of UCP-2 could be a viable strategy for the treatment of heart failure. 

The mechanisms underlying the sex-specific cardiac remodeling induced by pressure overload are addressed by García et al. in patients with aortic stenosis (AS) and mice subjected to transverse aortic constriction (TAC) [3]. This sexual dimorphism, characterized by a more fibrotic response in males but more hypertrophic in females, is associated with a sex-specific regulation of miR-29b in the myocardium under pressure overload. Furthermore, in women with AS preoperative plasma, the expression of miR-29b paralleled the severity of hypertrophy and was a predictor of adverse remodeling after aortic valve replacement. This article addresses a specific aspect of a very topical issue of how gender differentially affects the development, clinical symptoms, prognosis and responses to treatment in CVD [4].

The role of oxidative stress in cardiac alterations associated to obesity is the subject of the study by Jiménez González et al. [5]. The authors use a model of diet-induced obesity and myoblasts in culture to study the deleterious effects of high fat diet on cardiac function, and to show that antioxidants specifically targeted to mitochondria reduced cardiac oxidative stress and prevented cardiac damage. Sildenafil, an inhibitor of phosphodiesterase 5A (PDE5A), was also able to decrease the production of reactive oxygen species (ROS), fibrosis and inflammation, thereby recovering burn-induced cardiac dysfunction in a rat model [6].

Matilla et al.’s study also had implications in heart failure and the cellular effects induced by soluble interleukin 1 receptor-like 1 (sST2) [7], one of the promising new biomarkers in heart failure [8]. This molecule is a shed fraction of the interleukin 1 receptor-like 1 protein, a receptor of IL33, and is important for the inflammatory response. The authors find that cultured human cardiac fibroblasts are targets of sST2, having a profibrotic effect that is prevented by NFkB inhibitors. The critical role played by cardiac fibroblasts in chronic heart disease is reflected in the number of studies of the volume that address their biological functions [5,7,9,10,11]. For instance, the study by Martin et al. demonstrates that yet another mediator of inflammation, soluble phospholipase A2IIA (sPLA2-IIA), promotes myofibroblast differentiation and acts as a link between inflammation and fibrosis [9].

The intricacies of the mechanisms involved in the fibrotic response are clearly illustrated in the study by Valls-Lacalle et al. [10]. Here, the authors focus on connexin 43 (Cx43), a molecule essential for cardiac electrical coupling, with controversial effects on cardiac fibrosis, in fibroblast-mediated scarring. They dissect the role of Cx43 in a model of cardiac hypertrophy induced by angiotensin II, finding that contrasting effects could be found depending on the gene dose. This study analyzed several fibrotic markers, including lysyl oxidase (LOX), a major player in the fibrotic response to a cardiac insult and prototype of a family of extracellular matrix (ECM)-modifying enzymes essential for collagen cross-linking—an issue that is reviewed by Rodriguez et al. in the present volume [11].

Another cell surface receptor that yields a soluble form involved in cardiovascular pathophysiology is endoglin, a glycoprotein primarily expressed by the vascular endothelial cells. Gallardo Vara et al. study the relationship of soluble endoglin (sEng) with hypertension in preeclampsia, and using a combination of cellular and animal models find BMP4 as a downstream mediator of sEng effects [12]. Once again, these results show that circulating forms from proteolytically processed membrane-bound proteins are common in cardiovascular pathology, and can be useful as biomarkers to monitor the activation of key pathophysiological pathways involved in disease progression [13].

Endothelial cells not only are players in CVD, but are also a therapeutic option [14]. Poh et al. [15] explore the effects of thymosin-β4 (Tβ4) on endothelial progenitor cells (EPCs) in vitro, and in vivo in a diabetic rat model subjected myocardial infarction (MI). In the same field, Kutikhin et al. [16] provide an interesting characterization of endothelial colony-forming cells and its similarity with mature endothelial cells, a topic that has attracted a lot of attention recently, with the publication of several detailed maps of endothelial cell populations that underscore both the versatility of these cellular type and its implications on disease [17]. In a similar line, the study of Alexandru et al. found that cell-derived microvesicles, but in particular those derived from EPCs, protect against accelerated atherosclerosis induced in hamsters, by simultaneous exposition to hypertension and hyperlipidemia [18]. 

While most of the works mentioned so far center on cardiac diseases, several studies in the volume also explore pathologies specific of the vasculature. For instance, Park et al. found that gastrin releasing peptide (GRP) diminished calcification in cultured vascular smooth muscle cells (VSMC), and its inhibition reverted the phosphate-induced calcium deposition in rodent models [19]. By using models of accelerated aging, Del Campo et al. [20] identified VSMC as the main cellular type causing contractile impairment in a mouse model of Hutchinson-Gilford progeria syndrome (HGPS), and reported the beneficial effect of nitrite treatment. The impairment of vascular contractility is a part of the structural and functional alterations of the cardiovascular system in both physiological and premature aging, which in progeria patients, leads to accelerated arterial stiffness and atherosclerosis, and exaggerated CVD [21]. A combination of genetic models and cell cultures were also used by Marqués et al. for understanding the effect of NOX5-derived ROS in the activation of the COX-2/ prostaglandin E2 (PGE2) axis in endothelial cells under ischemic conditions [22]. This effect might play a relevant role in the pathophysiology of heart infarction and could be a potential therapeutic target.

The mingling of pathological mechanisms affecting cardiovascular health is clearly reflected by the risk of CVD associated with metabolic diseases, including conditions such as obesity or diabetes. This connection is explored in several studies of the present volume [5,15]. A link of these factors is studied by Actis-Dato et al. in their study of the involvement of LRP-1 in insulin response in a myoblast cell line [23]. The authors find that aggregated low-density lipoproteins (aggLDLs) inhibit the pro-signaling effect of LRP-1 in the insulin receptor, indicating that aggLDLs could contribute to insulin resistance in certain cells. 

Macrophages and the way they respond to LDLs, and particularly to electronegative LDLs, modified LDLs with increased atherogenic potential, are the subject of Puig et al.’s study [24]. They show the high uptake capacity of these particles by macrophages and a proinflammatory effect that was superior to other lipoproteins. As mentioned earlier, the interconnection of innate immunity and inflammation are also a focus of attention in the present volume [8,9]. The potential impact on plaque stability and atherothrombosis of macrophages and VSMCs expressing NOD1 (nucleotide-binding oligomerization domain-containing protein 1) is explored by González-Ramos et al. [25]. NOD1, a receptor that recognizes bacterial molecules and triggers an immune response, is activated by modified LDLs and over-expressed in atherosclerotic plaques [26], which has recently attracted the attention of vascular biologists investigating the relationship between inflammation/immune response and CVD. The implication of adaptive immunity in atherosclerosis is the subject of the study of Poels et al. [27], where T-cell activation due to an anti-cytotoxic T-lymphocyte associated protein 4 (CTLA4) treatment aggravates plaque progression. This could have important clinical implications, as anti-CTLA4 is an emergent therapy in certain cancers. Furthermore, specific aspects of the interconnection of the inflammatory machinery and cardiovascular disease are presented in two revisions on tumor necrosis factor-like weak inducer of apoptosis (TWEAK) [28] and bone morphogenetic protein 7 (BMP-7) [29], which exemplify how the innate immunity and the mechanisms of response to damage interact in the cardiovascular milieu.

Singla et al. also study M2 macrophage response in cardiac remodeling associated to the cardiotoxicity produced by the antineoplastic agent doxorubicin [30]. They found that embryonic stem cells, as well as exosomal derived from these cells increased M2 macrophages and anti-inflammatory cytokine and improved heart function ameliorating the cardiotoxicity of the drug. This finding could have potential implications in cardiac cellular therapies, but it also highlights the importance of a worrying aspect: the impact of cancer therapies on CVD due to the cardiotoxicity of antineoplastic drugs. 

Meeuwsen et al. tested neutrophils as biomarkers of subclinical coronary artery disease (CAD) in a high-risk population of women with a history of preeclampsia [31]. Neutrophils play a role in vascular damage in the placenta, as well as supporting the chronic inflammatory state in women with preeclampsia. However, the authors found no differences in the total number of neutrophils or their activation state, suggesting that these parameters are not directly associated with silent CAD disease burden and as such are not suitable biological markers of this medical condition. The study carried out in a cohort of women with a 10-20 years history of early onset preeclampsia illustrates the importance of registering and publishing “negative” results, especially in the context of clinical research in which, besides the study size, multiple characteristic factors of the specific population studied are essential. 

Finally, lymphocytes have also been visited in the present volume. An interesting work by Wernly et al. studied the effect of anti-CD3 antibodies in models of acute myocardial infarction (AMI) [32]. The study found decreased scar size after AMI, whereas cardioprotective and pro-angiogenetic pathways were unaltered by the treatment, qualitative changes in miRNA expression in extracellular vesicles (EVs) could be observed, illustrating new mechanisms through which cellular immunity plays a role in the cardiac healing process after an acute insult.

The present Special Issue aims to provide examples of the very rich field of cellular studies connected to cardiovascular research. Studies involving fibroblasts, endothelial cells, VSMC, cardiomyocytes, and leukocytes, among others, are included. Pathologies from myocardial infarction to preeclampsia have been investigated with a variety of cellular and animal models of disease. We wanted to examine the cellular mechanisms that lie behind the processes of repair and recovery of functionality after tissue damage in the context of CVD. Interestingly, most of the contributions proposed a target or mechanism that could be explored for therapy [2,5,6,7,9,12,15,25]. We have to be personally satisfied with the outcome, and we hope that you will find it also useful for your research. To finish, we would like to acknowledge all contributors, the editorial team at MDPI, and especially the scholars that took the time and effort needed to reviewing the manuscripts. It has been our pleasure to have you aboard, and we hope that it has been a delightful journey for you too!
